# Management of Chemoresistant and Quiescent Gestational Trophoblastic Disease

**DOI:** 10.1007/s13669-013-0071-6

**Published:** 2014-01-04

**Authors:** Siew-Fei Ngu, Karen K. L. Chan

**Affiliations:** Department of Obstetrics and Gynecology, The University of Hong Kong, 102 Pokfulam Road, Hong Kong, Hong Kong SAR

**Keywords:** Gestational trophoblastic disease, Gestational trophoblastic neoplasia, Chemoresistant, Quiescent gestational trophoblastic disease, Human chorionic gonadotropin, Hyperglycosylated human chorionic gonadotropin

## Abstract

Gestational trophoblastic neoplasia (GTN) is highly chemosensitive and has a high cure rate. Since the introduction of chemotherapy, reliable measurement of human chorionic gonadotropin (hCG) levels, and individualised risk-based therapy into the management of GTN, almost all low-risk and more than 80 % of high-risk GTN cases are curable. However, approximately 25 % of high-risk GTN developed resistance to chemotherapy or relapsed after completion of initial therapy, which often necessitate salvage combination chemotherapy. On the other end of the spectrum, a proportion of patients with gestational trophoblastic disease (GTD) have persistently low levels of hCG, without clinical or radiological evidence of disease, a condition called quiescent GTD. Recently, measurement of hyperglycosylated hCG has been proposed for the management of patients with quiescent GTD. Although representing a small proportion of GTD cases, the management of patients with chemoresistant and quiescent GTD often poses challenges to medical practitioners.

## Introduction

Gestational trophoblastic neoplasia (GTN) is highly chemosensitive and has a high cure rate. Since the introduction of chemotherapy, reliable measurement of human chorionic gonadotropin (hCG) levels, and individualized risk-based therapy into the management of GTN, almost all low-risk and more than 80 % of high-risk GTN cases are curable [1]. However, approximately 25 % of high-risk GTN patients developed resistance to chemotherapy or relapsed after completion of initial therapy, which often necessitate salvage combination chemotherapy [2•]. On the other end of the spectrum, a proportion of patients with gestational trophoblastic disease (GTD) have persistently low levels of hCG, without clinical or radiological evidence of disease, a condition called quiescent GTD [1]. Recently, measurement of hyperglycosylated hCG has been proposed for the management of patients with quiescent GTD [3]. Although representing a small proportion of GTD cases, the management of patients with chemoresistant and quiescent GTD often poses challenges to medical practitioners.

## Chemoresistant Gestational Trophoblastic Disease

Chemoresistant GTN occurs when there is a plateau or an increase in hCG levels, with or without development of new metastases, often while the patient is receiving therapy. Relapsed GTN occurs when there are at least two elevated levels of hCG in the absence of pregnancy after achieving a period of normal hCG values with treatment. Drug resistance and relapse are known to occur in around 3 % of low-risk GTN and 7-10 % of high-risk GTN cases [4].

Risk factors that predispose a patient to drug resistance and relapse include number of consolidation courses administered, clinicopathologic diagnosis of choriocarcinoma, initial hCG level, extent of disease (brain, liver, and gastrointestinal metastases have a worse prognosis), and higher World Health Organization (WHO) risk scores [5, 6]. Most of these patients are salvageable by further chemotherapy; however, 20 % of patients will eventually become resistant to treatment and die. The overall 5-year survival was more than 90 % for patients with relapsed GTN, which was nearly 100 % for low-risk GTN and about 85 % for high-risk GTN [7]. The prognosis for patients with chemoresistant GTN is worse than for those with relapsed GTN[7]. In a series of 81 patients, the long-term serologic complete remission rate reported was 52.6 % in chemoresistant GTN and 76.7 % in relapsed GTN [8].

### Management

For low-risk GTN patients who had been resistant to the single agent methotrexate, actinomycin-D is commonly used, followed by MAC (methotrexate, actinomycin-D, cyclophosphamide) or EMA-CO (etoposide, methotrexate, actinomycin-D, cyclophosphamide, vincristine) if further salvage therapy is needed [2•]. Methotrexate resistance was reported in about a third of patients with GTD where change of treatment was required. In a retrospective study of 485 patients who developed GTN after a hydatidiform mole, 150 (31 %) patients developed resistance to methotrexate [9]. Those patients who developed methotrexate resistance or toxicity at a relatively low hCG level (≤100 IU/L) were often cured with actinomycin-D, with a reported response rate of 87 %, while patients with hCG greater than 100 IU/L were salvaged with combination chemotherapy with EMA-CO, with a reported response rate of 99 % [9]. In the United Kingdom, actinomycin-D will replace methotrexate if hCG is less than 300 IU/l, while combined chemotherapy such as EMA-CO will be administered if hCG is higher than 300 IU/l [10]. In our centre in Hong Kong, actinomycin-D can be added for patients with low-risk GTN who do not respond to methotrexate.

For high-risk GTN patients who are resistant to first-line chemotherapy or have relapse, salvage combined chemotherapy with or without surgery will be required [11]. Various salvage regimens are used worldwide (Table 1); however, it is unclear which regimens are the most effective and the least toxic. Table 2 summarizes the salvage chemotherapy regimens reported in the literature (only publications after year 2000 are included) for chemoresistant or relapsed GTN. The favored regimen is EMA-EP (etoposide, methotrexate, actinomycin-D, etoposide, cisplatin) [2•].

**Table 1:** Salvage chemotherapy for resistant or relapsed gestational trophoblastic neoplasia

	*Chemotherapy Regimens*
EMA-EP	Etoposide, Methotrexate, Actinomycin-D, Etoposide, Cisplatin
BEP	Bleomycin, Etoposide, Cisplatin
TP/TE	Paclitaxel, Cisplatin / Paclitaxel, Etoposide
FA	5-Fluorouracil, Actinomycin-D
FAEV	Floxuridine, Actinomycin-D, Etoposide, Vincristine
MBE	Methotrexate, Bleomycin, Etoposide
VIP/ICE	Ifosfamide, Cisplatin, Etoposide

**Table 2:** Retrospective studies of salvage chemotherapy for resistant or relapsed gestational trophoblastic neoplasia

Authors, Year (Ref)	Primary Therapy	Salvage Therapy	Participants	Outcomes	Toxicity	Remarks
Newland et al. 2000 [47]	EMA-CO	EMA-EP	34 resistant/ relapsed	22 Non-assessable (hCG levels approaching normal) 12 Assessable: CR 75 %, OS 88 %	≥G3 neutropenia 68 % ≥G3 thrombocytopenia 40 %	
Matsui et al. 2002 [48]	MEA / EMA-CO	FA	10 resistant/ relapsed	OS 80 %	G4 leukocytopenia 6 % G4 thrombocytopenia 4 %	Mean FU 10 yr 1 had adjuvant surgery
Xiang et al. 2004 [49]	N/A	EMA-EP	12 resistant 3 PSTT	CR 11/15 (73 %) PR 3/15 (20 %)	N/A	
Ngan et al. 2006 [12]	CHAMOC / MA	MBE	A: 8 resistant B: 8 relapsed C: 4 ultra-high-risk	A: CR 88 %, 5-yr DFS 75 % B: CR 88 % C: CR 75 % Overall CR 85 %	≥G3 neutropenia 12 (60 %) ≥G3 thrombocytopenia 4 (20 %)	1 had adjuvant surgery 6 had brain radiation 1 had liver radiation
Wang et al. 2006 [50]	Various	MEF	9 resistant/ relapsed	CR 7/9 (78 %) OS 8/9 (96 %)	≥G3 neutropenia 26 % ≥G3 thrombocytopenia 5 %	Mean FU 37 mth
Mao et at. 2007 [51]	EMA-CO	EMA-EP	A: 11 resistant B: 7 relapsed	A: CR 9/11 (82 %) B: CR 3/7 (43 %) Overall CR 12/18 (67 %)	≥G3 neutropenia 28 % ≥G3 thrombocytopenia 3 % G3 hepatotixicity 3 %	8 had adjuvant surgery
Wan et al. 2007 [52]	Various	FAEV	11 resistant	CR 7/11 (64 %)	N/A	
Lu et al. 2008 [53]	EMA-CO	EMA -EP	10 resistant 3 relapsed	CR 11/13 (85 %)	N/A	5 had adjuvant surgery / brain radiation
Wang et al. 2008 [54]	Various	TP/TE	A: 16 relapsed (6 with failed cisplatin based regimen) B: 8 toxicities with previous therapy	A: Response rate 50 %: CR 3 (19 %), PR 5 (31 %) OS 44 % (70 % if 6 with failed cisplatin based regimen excluded) B: CR 2, PR 2, Non-assessable 4 OS 75 %	≥G3 neutropenia 10 (42 %) ≥G3 thrombocytopenia 3 (13 %) G2 renal toxicity 1 (4 %) Discontinued due to G3 neuropathy 1 (4 %)	A: Median FU 24 mth B: Median FU 19 mth
Zhao et al. 2009 [55]	FA	BEP	12 resistant	CR 10/12 (83 %)	N/A	
Feng et al. 2011 [56]	Various: mainly FAV, EMA-CO, 5-FU	FAEV	91 resistant/ relapsed	CR 55/91 (60 %) NR 29/91 (32 %) 3-yr OS 75 %	≥G3 neutropenia 24 (26.4 %) Febrile neutropenia 6 (6.6 %) ≥G3 thrombocytopenia 3 (3.3 %) Discontinued due to toxicity 7 (7.7 %)	23 of 55 SCR had adjuvant surgery
Lurain et al. 2012 [11]	EMA-CO	Various: EMA-EP; BEP; VIP; ICE; TP/TE	28 resistant	CR 82 %	N/A	11 had adjuvant surgery 4 had brain radiation
Manopunya et at. 2012 [57]	Various	FA	5 resistant (≥3 prior chemotherapy regimens)	CR 1/5 (20 %)	G4 neutropenia 25 % G3 diarrhoea 8 % G2/3 mucositis 92 %	

EMA-CO: etoposide, methotrexate, actinomycin-D, cyclophosphamide, vincristine; FA: 5-fluorouracil, actinomycin-D; CHAMOC: cyclophosphamide, hydroxyurea, actinomycin-D, methotrexate with folinic acid and vincristine; MA: methotrexate, actinomycin-D; MEA: methotrexate, etoposide, actinomycin-D; FAV: 5-fluorouracil, actinomycin-D, vincristine; 5-FU: 5-fluorouracil; EMA-EP: etoposide, methotrexate, actinomycin-D, etoposide, cisplatin; EP: etoposide, cisplatin; MEF: methotrexate, etoposide, 5-fluorouracil; BEP: bleomycin, etoposide, cisplatin; VIP: vincristine, ifosfamide, cisplatin; ICE: ifosfamide, cisplatin, etoposide; TP/TE: paclitaxel, cisplatin/paclitaxel, etoposide; MBE: methotrexate, bleomycin, etoposide; FAEV: floxuridine, actinomycin-D, etoposide, vincristine; PSTT: placental site trophoblastic tumour; hCG: human chorionic gonadotrophin; CR: complete response; OS: overall survival; PR: partial response; NR: no response; DFS: disease free survival; N/A: not available; FU: follow-up

Direct comparisons between the chemotherapy regimens in terms of outcomes and toxicity are inappropriate because the patients included were heterogeneous. Factors that influenced the salvage rate included type and number of previous chemotherapy regimens administered, use of adjuvant surgery or radiotherapy and WHO risk scores. Furthermore, the rates of toxicity reported are affected by the different measures used to counteract the toxic effects. For example, the threshold for administration of granulocyte-colony stimulating factor (G-CSF) will affect the rate of neutropenia reported.

The FIGO Cancer Report 2012 on trophoblastic disease suggested the use of EMA-EP protocol for patients resistant to EMA-CO or who have a recurrence after previous multiagent chemotherapy [1]. Alternatively, EMA (etoposide, methotrexate, actinomycin-D) may be used with cisplatin and doxorubicin. For EMA-EP resistant cases, TP/TE (paclitaxel, cisplatin / paclitaxel, etoposide) or paclitaxel and 5-fluorouracil, or ICE (ifosfamide, cisplatin, etoposide), or BEP (bleomycin, etoposide, cisplatin) have been used.

In our centre in Hong Kong, CHAMOC (cyclophosphamide, hydroxyurea, actinomycin-D, methotrexate with folinic acid and vincristine) has been used as first-line therapy for high-risk GTN, while MBE (methotrexate, bleomycin, etoposide) is used as second-line therapy. Our centre reported use of MBE in 16 patients who developed drug resistance to combination chemotherapy or relapsed after combination therapies, with a response rate of 88 % and 5-year disease free survival of 63 % [12].

The use of newer chemotherapy agents such as paclitaxel [13] and gemcitabine [14, 15], or high-dose chemotherapy with or without autologous bone marrow transplantation or peripheral stem cell support [16-19], may be considered in the management of selected high-risk patients. The major dose-limiting factor in most of these combined chemotherapy regimens is myelosuppression; therefore, most patients will require G-CSF and transfusion support to prevent treatment delays or dose reductions.

### Role of Surgery

Although GTN is chemosensitive, surgery may be required and can result in a cure in selected patients with chemoresistant or persistent foci of disease in the uterus or metastatic sites. During the course of treatment, about half of high-risk patients will require some form of surgical procedure to achieve cure [20].

The Charing Cross Gestational Trophoblastic Disease Center, UK, reported using hysterectomy in 9 of 20 patients who developed resistance to EMA-CO after other chemotherapy [21]. The Sheffield Trophoblastic Disease Center, UK, reported that 9 (75 %) of 12 patients who underwent hysterectomy for chemoresistant uterine disease had a complete clinical response to surgery [22].

The John I. Brewer Trophoblastic Disease Centre, USA, reported on a series of 50 patients with high-risk GTN treated with EMA-CO as primary or secondary therapy where 24 (48 %) had adjuvant surgical treatments, and 21 (87.5 %) were cured [23]. In a study by Lehman et al. involving 33 patients with chemorefractory GTD who underwent salvage surgery, 42 % had serologic complete response. 30 % had partial response, and 27 % had no response [24]. Initial salvage procedures included 29 hysterectomies, 4 thoracotomies, and 1 nephrectomy (in conjunction with a hysterectomy). A second salvage surgery was also performed in 4 patients, with all achieving complete response.

Factors that have been found to influence the therapeutic response to surgical interventions included age, type of antecedent pregnancy, preoperative hCG level, time from diagnosis to surgery, number of preoperative disease sites, preoperative WHO risk score, and histologic type [24, 25].

In cases where drug resistance is related to pulmonary metastasis and the lung lesion is amenable to operation, thoracotomy and lung resection, with a reported remission rate of up to 90 %, may be considered [26]. Criteria that have been used in patient selection for pulmonary resection included solitary pulmonary nodule, no evidence of other metastatic sites or uterine disease, and hCG level <1000 IU/L. Mutch et al. reported that 4 of 9 patients (44 %) who underwent thoracotomy with pulmonary wedge resection of resistant choriocarcinoma survived [27]. Patients with rapid regression of hCG within one to two weeks of surgical resection usually have a favorable outcome.

## Quiescent Gestational Trophoblastic Disease

Quiescent, or inactive, GTD occurs in a proportion of patients where there is a persistently low level of hCG in the absence of any clinical or radiological evidence of GTN. Usually the hCG level is in the range of 50–100 mIU/mL and remains static for at least 3 months [1, 28]. It is associated with prior history of GTD or spontaneous abortion, and does not respond to therapy [29-31]. This condition is thought to occur when a small focus of (or maybe individual) dispersed, differentiated syncytiotrophoblast cells are present. These slow-growing syncytiotrophoblast cells produce small stable amounts of hCG and do not usually progress to invasive disease as long as the cytotrophoblast, or intermediate cells, are absent [32]. These syncytiotrophoblast cells do not respond to chemotherapy, and surgery does not result in normalization of hCG [31].

### False-Positive hCG

Quiescent GTD must be differentiated from false-positive hCG test results, or so-called “phantom hCG”. Approximately 2 % of women of reproductive age will have a low level of hCG (<300 mIU/mL) detectable by conventional hCG test without the presence of trophoblasts [33]. The false-positive results have led to some women being diagnosed with GTD and undergoing various diagnostic procedures, chemotherapy, hysterectomy, and other surgical procedures before it is established that the results are spurious.

This false-positive test result occurs as a result of the presence of nonspecific heterophile antibodies in the patient’s serum, which binds the animal antibodies used in the hCG assay [34]. False-positive hCG can be determined by 1) urine hCG assay, which will be negative because heterophile antibody is not excreted in the urine due to the large molecule size; 2) serial dilution of the sample, as hCG levels are unaffected by dilution; 3) use of different commercial assays that will often result in a significant fluctuation in the hCG level [35].

False-positive hCG test results can also be found in 1 % of perimenopausal and 7 % of postmenopausal women. These false-positive results are due to raised levels of pituitary follicle-stimulating hormone and luteinizing hormone, as well as a benign low level of pituitary hCG secretion [36]. In the case of pituitary hCG, the production can be inhibited with oral contraceptive pills [37]. Treatment is not required in false-positive hCG tests, because an abnormal trophoblast is absent.

### Hyperglycosylated hCG

Hypergylcosylated hCG (hCG-H) measurement has been proposed for the management of patients with quiescent GTD [3]. hCG-H is a glycoprotein produced by cytotrophoblast cells and is associated with trophoblast invasion, growth of cytotrophoblast cells, and overall promotion of placental implantation [32, 38-40]. It is a promoter of choriocarcinoma growth and tumorigenesis, and is the main form of hCG produced in active choriocarcinoma and gestational trophoblastic neoplasm [41].

The USA hCG Reference Service has demonstrated that the proportion of hCG-H (hCG-H / total hCG) is a 100 % sensitive marker for distinguishing active GTN/choriocarcinoma from quiescent GTD and suggested its use as a marker to identify active trophoblastic malignancy [42]. In their study comparing the proportion of hCG-H in 82 women with GTN (including 30 with histologic choriocarcinoma), 26 with resolving hydatidiform mole and 69 with quiescent GTD, the proportion of hCG-H was found to be significantly higher in GTN/choriocarcinoma cases than those with resolving hydatidiform mole or quiescent GTD [42].

Quiescent GTD is likely the most common cause for persistently low hCG levels outside of pregnancy in women of reproductive age. hCG-H has been found to be undetectable or at very low levels in patients with quiescent GTD, and therefore is useful for their identification. In a report of 133 patients diagnosed with quiescent GTD, all had low hCG levels persisting for 3 months or longer and a history of GTD [3]. Of these patients, 127 (95 %) had undetectable hCG-H, and 6 had low positive hCG-H, accounting for 4–27 % of serum total hCG concentration. In this condition, chemotherapy was ineffective because the tissue in quiescent GTD is not growing, and in most cases hCG returned to normal within 6 months. Thus, it was suggested that when hCG-H is undetectable, even with persistently low hCG levels, intervention is not needed. Meanwhile, if hCG-H becomes detectable, then this may indicate clinically relevant disease and therapy may be required.

Approximately 20 % of patients with quiescent GTD will start to produce increased hCG after a period of several weeks to years [1, 42]. During the quiescent period, the hCG-H is undetectable, but once the hCG rises, a significant proportion is hCG-H; this is often noted before the appearance of clinically detectable disease. Furthermore, hCG-H were able to first detect active disease 0.5 to 11 months prior to rapidly rising hCG or clinically detectable tumour [42]. Hence, hCG-H has been suggested as a marker for the early detection of new or recurrent GTN/choriocarcinoma.

### Management

The International Society for the Study of Trophoblastic Disease 2001 recommended that in the management of patients with quiescent GTD, false-positive hCG results should be ruled out and that investigations for evidence of disease should be performed. Immediate chemotherapy or surgery should be avoided, and long-tem monitoring with serial hCG while avoiding pregnancy should be advised [43]. In the event that there is significant rise in hCG or presence of overt clinical disease, then treatment should be instigated promptly.

Recently, Agarwal et al. at the Charing Cross Gestational Trophoblastic Disease Center reported on 76 patients from a cohort of 13,960 with hydatidiform moles who had persistently elevated but declining hCG levels 6 months after evacuation [44•]. In this study, 66 (87 %) patients were treated expectantly, where 65 (98 %) had spontaneous resolution of hCG to normal and 1 had persistently elevated hCG due to chronic renal failure, but she remains healthy. The duration for hCG to return to normal was within 8 months of evacuation for 44 (67 %) patients, in the next 4 months for 15 (23 %) patients, and longer than 1 year for 6 (9 %) patients. The remaining 10 (13 %) were treated with chemotherapy, and hCG returned to normal in 8 (80 %) of these patients, but remained slightly raised, though asymptomatic, for 2 (20 %) patients. Argarwal concluded that surveillance for more than 6 months is safe for women with persistently high but falling hCG levels, because a declining trend represents spontaneous, although slow, regression of residual molar tissue.

The findings of the study by Argawal et al. are reassuring; however, how should a decision on whether a patient be observed or treated with chemotherapy be made? The investigators suggested a cut-off hCG level of 345 IU/L at 6 months – which was the median hCG value of patients who responded to chemotherapy in their cohort – for initiating chemotherapy [44•]. Meanwhile, Cole et al. suggested that in quiescent GTD, chemotherapy should be initiated only when hCG begins to rise and is >3000 IU/L, because chemotherapy would probably be ineffective below this level [3]. However, adopting this approach could lead to progression of disease to a more advanced stage, including development of distant metastases, which is associated with unfavorable prognosis and survival [45].

Utilization of hCG-H could improve the management of quiescent GTD and help to identify the characteristics of the condition before starting treatment. However, this approach is only feasible if the hCG-H assay is readily available and affordable. Furthermore, its use needs to be validated by gestational trophoblastic disease management centres [46]. Therefore, it is essential that treatment be individualized, and preferably patients with GTN should be managed in centres with dedicated specialists. Centres with many patients should collaborate to enable data collection and establishment of reasonable hCG cut-off values, and ultimately improved management of this small but intriguing group of patients.

## Conclusion

Ideally, patients with chemoresistant, or quiescent, GTD should be managed at a Trophoblastic Centre. A single-center, randomized controlled trial comparing interventions for chemoresistant, or quiescent, GTN will be very challenging, given the small numbers of patients with these conditions. Therefore, international multi-center collaboration is required to provide the high-quality evidence required to determine the most effective treatment.
